# Genomics of the Parasitic Nematode *Ascaris* and Its Relatives

**DOI:** 10.3390/genes12040493

**Published:** 2021-03-28

**Authors:** Jianbin Wang

**Affiliations:** 1 Department of Biochemistry and Cellular and Molecular Biology, University of Tennessee, Knoxville, TN 37996, USA; jianbin.wang@utk.edu; 2 UT-Oak Ridge National Laboratory Graduate School of Genome Science and Technology, University of Tennessee, Knoxville, TN 37996, USA

**Keywords:** *Ascaris*, parasitic nematode, genome, transcriptome, small RNA, histone modification, chromatin, comparative genomics, chromosome, evolution

## Abstract

Nematodes of the genus *Ascaris* are important parasites of humans and swine, and the phylogenetically related genera (*Parascaris, Toxocara*, and *Baylisascaris)* infect mammals of veterinary interest. Over the last decade, considerable genomic resources have been established for *Ascaris*, including complete germline and somatic genomes, comprehensive mRNA and small RNA transcriptomes, as well as genome-wide histone and chromatin data. These datasets provide a major resource for studies on the basic biology of these parasites and the host–parasite relationship. *Ascaris* and its relatives undergo programmed DNA elimination, a highly regulated process where chromosomes are fragmented and portions of the genome are lost in embryonic cells destined to adopt a somatic fate, whereas the genome remains intact in germ cells. Unlike many model organisms, *Ascaris* transcription drives early development beginning prior to pronuclear fusion. Studies on *Ascaris* demonstrated a complex small RNA network even in the absence of a piRNA pathway. Comparative genomics of these ascarids has provided perspectives on nematode sex chromosome evolution, programmed DNA elimination, and host–parasite coevolution. The genomic resources enable comparison of proteins across diverse species, revealing many new potential drug targets that could be used to control these parasitic nematodes.

## 1. Introduction

*Ascaris* is a large nematode (roundworm) that lives in the small intestine of its hosts, pigs and humans [1]. It infects ~800 million people, mostly children [2,3,4]. *Ascaris* infection, known as ascariasis, contributes significantly to global disability-adjusted life years, perpetuating the cycle of poverty in areas of endemic infection, particularly in parts of Africa, Southeast Asia, and South America [5,6,7]. Historically, two species of *Ascaris* that infect humans (*Ascaris lumbricoides*) or pigs (*Ascaris suum*) have been described, but cross-infections are observed [8,9,10] and these two species have little difference in anatomy, physiology, and genome sequences [11]. Thus, for simplicity, *Ascaris* refers to both species in this review. *Ascaris* and its relatives have been the subject of many basic scientific studies, leading to the Boveri–Sutton chromosome theory of inheritance [12,13] and insights into programmed DNA elimination (also known as chromatin diminution) [14,15,16], trans splicing [17,18,19,20], neurobiology [21,22], metabolism [23,24,25,26], and amoeboid sperm in nematodes [27,28,29,30].

*Ascaris* is well-suited for biochemical and genomic studies due to the size of adults (on average 30 cm for females and 15 cm for males) and the large numbers of eggs produced [1]. This allows easy isolation of large amounts of material from distinct developmental stages, including various germline and somatic tissues. *Ascaris* is sexually dimorphic, and the male and female reproductive systems are about 1 to 2 m in length when fully extended, enabling dissecting and collecting of the mitotic germline, transition zone, and all stages of meiosis, including mature oocytes and spermatids [1]. For example, one can isolate 20–40 μL of packed spermatids from a single seminal vesicle. In addition, a single female is estimated to release 0.2 to 1 million eggs per day and the uterus (~20–25 cm in length) can hold up to 27 million eggs [31,32]. The fertilized eggs can be easily isolated. They undergo synchronized development under controlled lab conditions, allowing the collection of hundreds of millions of embryos at discrete stages of development, as well as larvae, for in vitro biochemical [17,20,33], cellular, molecular, and genomics studies [34,35,36,37,38,39].

The development of genomics technologies and the decrease of sequencing costs have made genome sequencing, transcriptome profiling, ChIP-seq, and comparative genomics feasible in individual labs. In the last decade, many large-scale genomics studies have been carried out on *Ascaris* and its relatives (Table 1). This led to a rich trove of genomic data that has provided key insights into many biological questions, such as the function and mechanism of programmed DNA elimination [35,37,38,39], the contribution of transcription to early animal development [36,40], and the diversity of small RNAs in nematodes [34,41,42,43,44,45]. The studies have also provided genomics resources for identifying potential drug targets [46,47,48,49,50,51] and worm control [9,11,52,53,54,55]. In this review, I summarize the development of current *Ascaris* genomic resources, including the complete germline genome and a comparison to the somatic genome, transcriptomes and small RNAs, epigenomes, and chromatin accessibility datasets. I also discuss population genomics, as well as comparative genomics and chromosome evolution in *Ascaris* and its related nematodes.

## 2. History of *Ascaris* Genome Assemblies

The *Ascaris* genome was studied extensively before the development of modern genomic approaches. One particular interest was to determine the amount of sequence lost and identify the eliminated DNA and the sites of DNA breaks during programmed DNA elimination [14,15]. Using biochemistry and chromosome staining, the germline genome was estimated to be 300 to 800 Mb by different research groups [56,57,58,59] and an estimated ~20–40% of DNA was suggested to be lost during DNA elimination. Through molecular cloning and sequencing, the nature of eliminated DNA began to be unveiled, with the first identified element as an abundant 121-bp tandem repeat [60,61]. Later, other minor repeats [62] as well as three protein-coding genes [63,64,65] were found to be eliminated. It was also established that the DNA break ends are healed by telomere addition during DNA elimination [66,67,68].

At the turn of the 21st century, first-generation high-throughput Sanger sequencing was used to generate many high-quality reference genomes, including those of important human parasites [69,70,71,72,73,74,75,76,77]. However, the cost to generate a de novo genome assembly was still very expensive. Although being a parasite infecting upwards of 800 million people, *Ascaris* was not promoted as a priority to be sequenced at large genome sequencing centers mainly due to the small number of researchers working on *Ascaris* and its relatively large genome size. Indeed, before next-generation sequencing technologies, there were only ~1× coverage of Sanger and ~3× coverage of 454 *Ascaris* genomic reads available. The first significant *Ascaris* draft genome was generated using the Sanger and 454 reads together with ~14× Illumina reads [34] (Table S1). The draft genome and the extensive transcriptomes were mainly used to facilitate small RNA data analysis [34]. Soon after, two additional draft assemblies using high coverage of Illumina reads were published (Table S1), one focused on genomics resources for drug discovery [46] and the other comparing the germline and somatic genomes for programmed DNA elimination [35].

**Figure 1:**
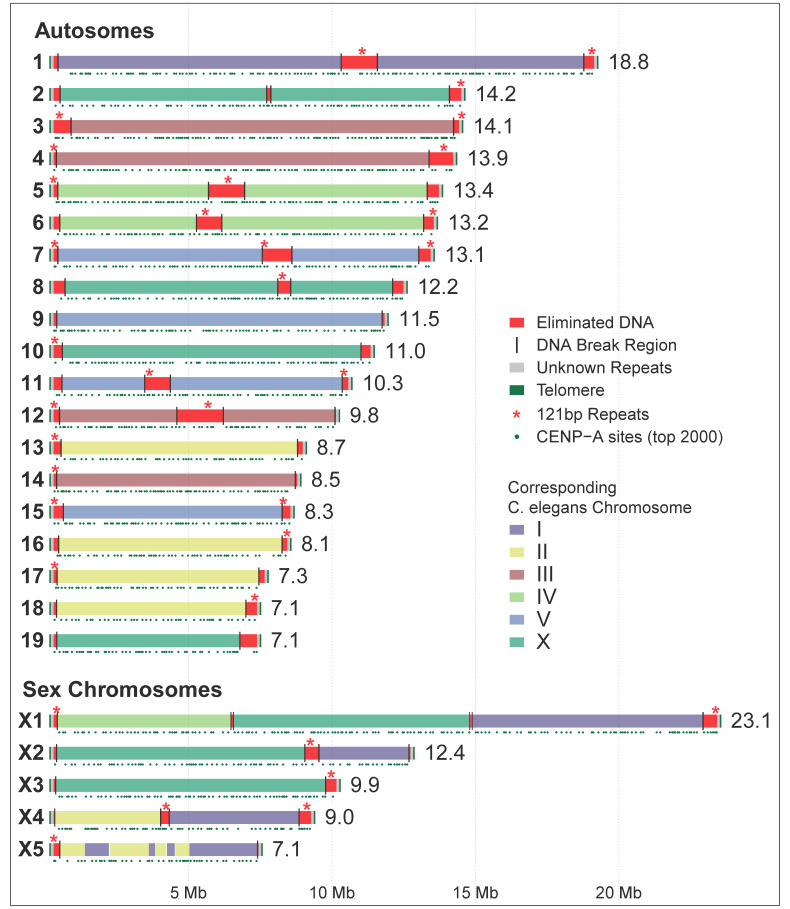
*Ascaris* genome, programmed DNA elimination, and chromosome evolution. Germline chromosomes are illustrated with the length of the chromosome in Mb on the right. Chromosome regions that are eliminated are colored in red. The retained regions, which will become somatic chromosomes after DNA elimination, are colored based on their corresponding *C. elegans* chromosomes (see legends). CENP-A deposition (data from 12 developmental stages [37]) are illustrated by green dots underneath the chromosomes, illustrating possible centromeric regions for the holocentric chromosomes; shown are the top 2000 (out of 3342) CENP-A enriched sites.

The germline and somatic assemblies were further improved with new technologies, including long reads, optical mapping, and Hi-C, to produce two major recent updates (Table S1) [38,39]. The latest *Ascaris* germline and somatic genomes represent fully assembled chromosomes [39], with 24 germline chromosomes and 36 in somatic cells; all repetitive sequences were anchored in these assembled chromosomes (Figure 1). 

Along with these genomes, extensive transcriptomes, small RNA datasets, and epigenomes have been produced that provide comprehensive genomic data for *Ascaris*. In addition, a comparison of *Ascaris* genomes from humans and pigs revealed that the two genomes are very similar, with extensive heterozygosity, existing as genetic mosaics, and thus reflecting the highly interbred nature of *Ascaris* species [11].

## 3. *Ascaris* Genomes

One of the most striking features of *Ascaris* is that the adult worms have two genomes: an intact germline genome and a reduced somatic genome. This reduced somatic genome is the result of a process called programmed DNA elimination, where parts of the germline genome are lost during the differentiation of germ cells into somatic cells in early embryogenesis [14,15,16]. This process occurs in five independent cell lineages between the 4–16 cell stages of development and is achieved through double-stranded DNA breaks in chromosomes followed by selective loss of portions of the chromosome fragments [35,37,38,39]. Comparison of the germline and somatic genomes suggest that there are 72 chromosomal break regions (CBRs), with 48 at the chromosome ends that removes all subtelomeric and telomeric sequences to form the somatic genome (Figure 1). The other 24 CBRs are in the middle of the chromosomes that contribute to an increased number of somatic chromosomes (Figure 1).

About 55 Mb (~18%) of the *Ascaris* germline DNA is eliminated in somatic cells during programmed DNA elimination. This includes ~30 Mb of a 121-bp tandem repeat and ~5 Mb of subtelomeric and telomere repeats. Strikingly, the other 20 Mb of eliminated DNA is unique and encodes ~1000 germline-expressed genes [35,38,39]. The majority of these eliminated genes are specifically expressed in the testes and therefore are likely involved in *Ascaris* spermatogenesis. The data suggests that DNA elimination is an alternative way of silencing these germline genes in the somatic cells; rather than repressing their expression, they simply remove them from the genome as a permanent way of gene silencing. The sequences lost and the break regions are the same in the five independent elimination events, in individual males and females, and in worms from pigs and humans [35,38,39]. This programmed and high-fidelity removal of DNA during early development suggests specific and regulated mechanisms are involved in *Ascaris* DNA elimination.

Another interesting feature of the *Ascaris* genome is that it has multiple sex chromosomes, with five in the germline and nine in the somatic cells. This is in contrast with most nematodes (including the model *C. elegans*) that have only one sex chromosome. Comparison of nematode chromosomes using orthologous proteins (reciprocal best blast hits) indicates that many of the *Ascaris* germline sex chromosomes are likely derived from recent chromosome fusion events (Figure 1) [39]. This is further supported by chromosome “painting” analyses using conserved Nigon elements [78]. These comparative analyses of nematode chromosomes revealed that while extensive intra-chromosomal rearrangements occurred, the karyotypic unit for nematodes remains largely intact except for occasional scission and fusion events [78]. Interestingly, the fused *Ascaris* germline sex chromosomes are broken at the sites of these fusions during DNA elimination, resulting in the pre-fusion karyotypes in somatic cells (Figure 1) [39]. In addition, Hi-C data suggests that the sex chromosomes in the testis have greater interactions with each other than with the autosomes [39]. This high level of interaction among the sex chromosomes suggests that they are likely physically close to each other during meiosis, reminiscent of the chromosome behavior during meiosis in other systems with multiple sex chromosomes [79,80,81,82]. More chromosomal assemblies from ascarids and other nematodes are needed to further study the evolution and interactions of nematode chromosomes.

## 4. *Ascaris* Genes and Transcriptomes

**Figure 2:**
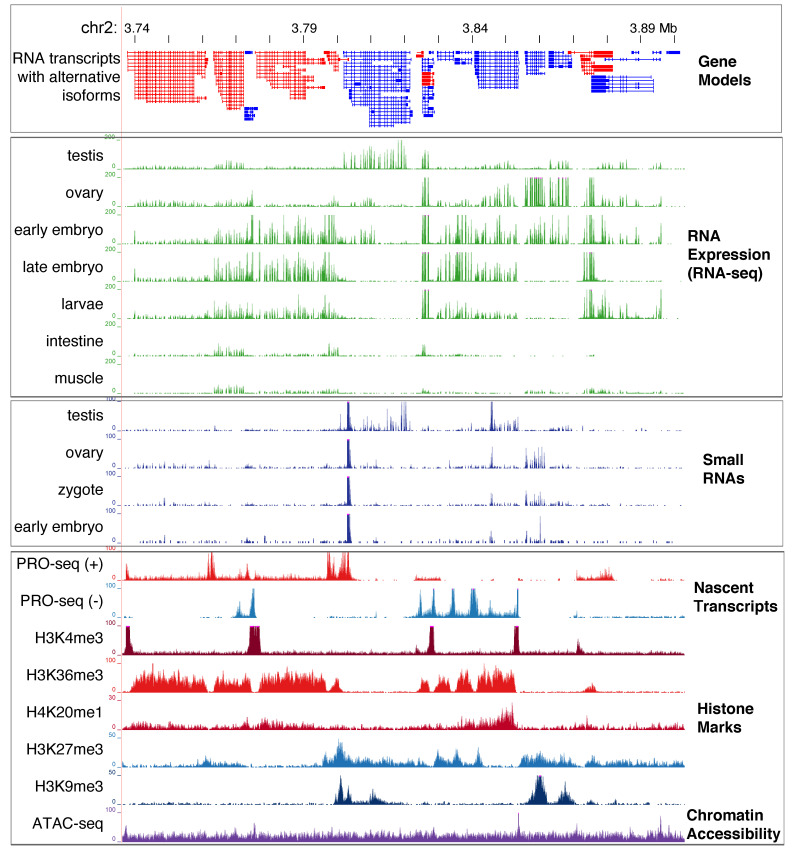
*Ascaris* genome data illustration. Genome browser tracks with gene models, RNA expression, small RNAs, histone marks, and other datasets are illustrated. For the gene models, RNA transcribed on the plus strand is in red and minus strand is in blue. The data for tracks on nascent transcripts, histone marks, and chromatin accessibility are derived from 32–64-cell (5-day) embryos. The genome browser can be accessed at http://genome.ucsc.edu/s/jianbinwang/Ascaris_genome_browser_genes_review, access on 24 March 2021.

The predicted number of genes for *Ascaris* genome varies from 15,000 to 19,000 depending on the genome assembly and transcriptome data (see Table S1). The differences are a consequence of completeness of the genome assemblies and the transcriptome datasets used for gene identification. Early draft genomes tend to predict higher number of genes due to split gene fragments and the presence of multiple uncollapsed contigs. More comprehensive and stage-specific transcriptomes enable assembly and the identification of tissue-specific genes. The latest genome version (v4, see Table 1 and Table S1) predicts 15,714 genes, with 918 genes eliminated by DNA elimination. This version used 42 RNA-seq datasets from various developmental stages, including distinct stages of the male and female germline, early zygotes, embryos, larvae, muscle, intestine, and other somatic tissues (Figure 2). Over 60,000 alternative spliced isoforms were identified across these stages. PacBio ISO-seq data (see available genomic resources for ascarids) were used to further refine and validate these transcripts. Like most other nematodes and flatworms, *Ascaris* mRNAs undergo trans-splicing and some genes are encoded in polycistronic RNAs [18,83,84]; thus, trans-splicing, genome assembly, and transcriptome data should be carefully examined and additional data (e.g., PCR) may be necessary for further curating the gene models. ChIP-seq data on histone modification marks, such as H3K4me3 and H3K36me3, can be used to further define the transcription start sites and the gene bodies; nascent transcription data, such as GRO-seq and PRO-seq, can better define the starts and stops of transcription units (Figure 2); and ribosome profiling [85] can improve annotation on protein-coding regions and also help distinguish mRNAs vs. long non-coding RNAs (lncRNAs).

RNA-seq through *Ascaris* early development has provided some novel insights into early gene expression and gene regulation during embryogenesis [36,40]. In most model organisms, the maternal to zygotic transition—the turnover of maternally contributed mRNAs and the activation of zygotic transcription—does not start until ≥2 cell number during embryogenesis [86,87,88]. However, RNA-seq data from *Ascaris* oocytes, sperms, zygote maturation, and early embryogenesis revealed ~4000 genes are transcribed prior to pronuclear fusion and in the 1–4 cell embryos [36]. Transcription prior to pronuclear fusion is in drastic contrast to most model organisms. The free-living nematode *C. elegans* has almost identical cell lineages and morphological patterns during early development. While major transcription is not active until late gastrulation and organogenesis (~100 cells) in *C. elegans*, many orthologous *C. elegans* maternally contributed RNAs are transcribed in *Ascaris* zygotes and early embryos [36]. Due to its rapid embryonic development, *C. elegans* largely relies on maternal regulators to drive early zygotic gene expression and embryogenesis. In contrast, *Ascaris* transcribes new RNAs as needed during early development. Thus, gene regulation in early development is largely transcriptional in *Ascaris*, whereas it is mainly regulated post-transcriptionally in *C. elegans*. The difference in the gene regulation programs is likely due to the differences in cell cycle length during early development. The extended maturation of zygotes prior to pronuclear fusion and long early cell cycles of *Ascaris* (15–20 h) provides ample time for the transcriptional machinery to make new RNAs [36,40]. Additional transcriptome analyses of early development in different nematodes are likely to yield insights into gene regulation in early development.

RNA-seq analyses from *Ascaris* intestines have facilitated the understanding of the nematode intestine and efforts to develop the intestine as a potential target for new drug therapies [45,89,90,91]. Multiomics across diverse nematode species, including mRNAs and miRNAs from *Ascaris*, were used to define and investigate the biology of the intestine. The large size of adult *Ascaris* allows easy dissection of anterior (close to head), middle, and posterior regions of the intestine, enabling the identification of differentially expressed transcripts in these regions. Analysis of the intestinal transcriptome shows many unique and highly expressed transcripts are present in the anterior region [45]. Together with proteomics [92] and the development of *Ascaris* intestinal cannulation and a perfusion model [93], an experimental system was established for testing and validation of drug candidates. The *Ascaris* intestine model represents an important system for developing novel anthelmintic drugs against nematode intestinal cells [91,94].

## 5. *Ascaris* Small RNAs

In addition to the protein coding mRNAs, transcriptomes also contain structural and regulatory RNAs, such as rRNAs, tRNAs, lncRNAs, as well as various types of small RNAs. The nematode *C. elegans* has three major classes of known small RNAs: miRNAs, siRNAs (22G-RNAs and 26G-RNAs) and piRNAs (21U-RNAs) [95,96]. These small RNAs interact with the genome, transcripts, or foreign sequences to silence or regulate their targets. In an early study, ~100 high-confidence *Ascaris* miRNAs as well as numerous siRNAs were identified [34]. Most of the siRNAs are 22G-RNAs with 5’-triphosphates that target repeats and/or mRNAs that are dynamically expressed throughout germline and embryonic development. *Ascaris* also has 26G-RNAs with 5’-monophosphates that are specific to the testis and only target testis-specific genes [34]. Surprisingly, piRNAs, Piwi-clade Argonautes, and other proteins associated with the piRNA pathway are lost in *Ascaris* [34]. This loss of the piRNA pathway was later found to be common in other nematodes [44]. Initial studies found no evidence to suggest that small RNAs may contribute to DNA elimination [34]. However, this could be due to the large number and complexity of total *Ascaris* small RNAs present and thus the inability to identify unique small RNAs associated with DNA elimination.

Small RNAs are associated with Argonaute proteins. While *C. elegans* has an expanded Argonaute family of 25 proteins [97], *Ascaris* has a reduced number of 10 Argonautes [34]. Most studies on *Ascaris* small RNAs have focused on miRNAs [41,42,45,52]. However, the predominant *Ascaris* small RNAs are endogenous siRNAs, estimated to be over 90% of all small RNAs in *Ascaris* and other parasitic nematodes. The role of these endogenous small RNAs in nematodes needs to be explored. In the parasitic nematode *Heligmosomoides*, an Argonaute and siRNAs were found in secreted extracellular vesicles that may play a role in host–parasite interactions [98,99]. Outstanding questions related to small RNAs in *Ascaris* and other nematodes include: (1) What specific small RNAs are associated with each Argonaute? (2) What are the biological roles of each Argonaute and its associated small RNAs? (3) Do any particular Argonautes and their associated small RNAs play a role in DNA elimination? (4) How do small RNA contribute to host–parasite interactions and transgenerational epigenetic inheritance? And (5) how do *Ascaris* and other nematodes compensate for the loss of piRNAs?

## 6. *Ascaris* Chromatin and Epigenome

In eukaryotes, a common mechanism of gene regulation is through the differential organization of nucleosomes and chromatin, forming heterochromatin or euchromatin. Chromatin status can be associated with histone modification marks that activate or repress specific genomic regions [100]. In *Ascaris*, antibodies against many common histone modifications have been used in immunohistochemistry, electron microscopy, and ChIP-seq [37,38,39]. ChIP-seq data are available for common modifications, such as the active marks H3K4me3, H3K36me3, and H4K20me1, as well as the repressive marks H3K27me3 and H3K9me3 in a few developmental stages (Figure 2). These data provide initial insights into the *Ascaris* epigenome. They can also be used to further curate gene annotation and facilitate gene expression and other genomic analysis. Future data on the changes of histone marks through *Ascaris* development may also help elucidate the nature of epigenetic inheritance during gametogenesis, fertilization, and early embryogenesis in this parasitic nematode.

The accessibility of *Ascaris* chromatin during early development was also evaluated using ATAC-seq [38]. ATAC-seq uses a Tn5 transposon to insert into regions of the genome that are more open, thus providing a measurement of DNA accessibility [101]. The sites of *Ascaris* chromatin accessibility are enriched at the promoter regions of active genes, where H3K4me3 is also enriched (Figure 2). Interestingly, *Ascaris* chromosomal break regions (CBRs) for DNA elimination become more accessible just prior to DNA elimination (4-cell stage at 60 hour) compared with earlier embryo stages (0 hour) and germline tissues, with the open regions matching where new telomeres are added to the broken DNA ends [38,39]. This more accessible chromatin in the CBRs might be due to a reduced number or compactness of nucleosomes or other epigenetic changes. It is plausible that factors, such as DNA replication stress, RNA transcription, R-loops, and/or the 3-D organization of the chromosomes, might lead to the more open chromatin at these CBRs. These factors and processes might also recruit the telomere addition machinery to the regions, further opening the CBRs in the subsequent cell cycles after elimination [38,39].

An epigenetic mark for centromere deposition is the variant of histone H3 called CENP-A [102,103,104]. Unlike its hosts (pig and human) that have monocentric chromosomes with one centromere per chromosome, *Ascaris* has holocentric chromosomes with many centromeres distributed along the length of the chromosomes. This is consistent with observations in the model nematode *C. elegans* [105,106]. ChIP-seq using specific antibodies against *Ascaris* CENP-A (and CENP-C) identified many enriched loci with these marks across the length of the chromosomes (Figure 1). Deposition of CENP-A is not associated with repetitive or any specific sequences, is inversely related with transcription, and the pattern of deposition changes through gametogenesis and embryogenesis [37]. Interestingly, *Ascaris* CENP-A is enriched in all regions in both male and female germline, but its level is reduced prior to and during a DNA elimination mitosis in the eliminated regions. The loss of CENP-A and thus functional centromeres in the eliminated regions provides a mechanism for the selective loss of sequences after the DNA breaks during DNA elimination [37]. However, questions on what identifies the CENP-A deposition sites and how much CENP-A is required in nematodes to form a functional microtubule attachment site remain to be determined.

## 7. Population Genomics of *Ascaris* from Pigs and Humans

*Ascaris* has historically been considered to have two main species, distinguished by their host: *A. suum* from pigs and *A. lumbricoides* from humans. They are, however, known to cross-infect both hosts [8,9,10] and are capable of interbreeding [107,108]. Population genetic studies generally agree that the genetic differences in *Ascaris* collected from around the world are caused by geographic reproductive isolation [9,109]. Data from mitochondrial sequence analyses suggest there are human-associated and pig-associated clades in *Ascaris* [110,111] or multiple haplotype clusters [112]. A recent study using a reference-quality *A. lumbricoides* genome and 68 worms from human hosts in Kenyan villages identified over 11 million SNPs [11]. Comparative phylogenomic analyses of these SNPs indicates that these worm genomes had extensive heterozygosity with genetic mosaics, suggesting a highly interbred *Ascaris* species genetic complex. Analysis of the complete mitochondrial genomes from these 68 individual worms and other sequences available also supports the idea that worms from pigs and humans form a genetic complex that is capable of interbreeding [11]. This large-scale phylogenomic study on both the nuclear and mitochondrial genomes in *Ascaris* suggests recent and multiple cross-infection events occurred in *Ascaris* populations, likely caused by the immigration of humans and livestock. As *Ascaris* can infect both pigs and humans, a one-health approach is critical to control the spread of human ascariasis.

## 8. Comparative Genomics and Ascarids Evolution

Large parasitic nematodes like *Ascaris* are known as ascarids. A variety of ascarids are known to infect essentially all large mammals. Common genera include *Ascaris* (from pigs, sheep, monkeys, apes, and humans), *Parascaris* (from horses, donkeys, and zebras), *Toxocara* (from dogs, cats, tigers, and lions), *Baylisascaris* (from raccoons, pandas, bears, badgers, and marmots), and the more distantly related marine ascarids *Anisakis* (fish and marine mammals). Most of these parasites have relatively large (~300 Mb) genomes and chromosome number (>20) (Table 1), except for some species of *Parascaris*, one of which has a single chromosome and a huge 2.5-Gb genome (*P. univalens*) and another one with a much smaller genome (~130 Mb for *Anisakis simplex*). Adult ascarid infections in general cause mild symptoms in their hosts. However, heavy infections can lead to intestinal obstruction and sometimes death. Fatality is more common in horses and pandas.

A number of other ascarids genomes have recently been sequenced (Table 1). The *Toxocara canis* genome from dogs was used to identify genes for potential new drug targets and to provide a genomic resource for future molecular studies [49]. An improved version of this genome was generated using additional reads from the 50 helminth genomes initiative at the Sanger Institute that enabled characterization of DNA elimination in *Toxocara* [38]. The germline and somatic genomes from the horse parasite *Parascaris* were also sequenced (Table 1). Comparative analysis of *Ascaris*, *Parascaris*, and *Toxocara* genomes reveals that about 1000 to 2000 germline-expressed genes are eliminated, with 35% of these eliminated genes conserved among the genera that are preferentially expressed during spermatogenesis [38]. These data support the idea that DNA elimination is a way to silence these germline genes in the somatic cells. Testes are known to be the birthplace for many new genes [113,114,115,116]. In these ascarids, the removal and thus permanent silencing of the testis genes in the somatic cells may enable large sampling of new genes for reproduction, providing a mechanism for more rapid evolutionary changes in the testis [66] while their elimination in the soma prevents deleterious effects in other tissues. Additional ascarid genomes and transcriptomes will likely reveal new insights into the evolution of testis-specific genes and programmed DNA elimination. Recently, an analysis of the initial draft genome of the marine ascarids *A. simplex* infecting herring provided information on carbohydrate metabolism genes in the parasite’s development [117]. Future improved genomes and omic studies promise to shed lights on the genomics and molecular processes of these important marine parasites (see review [118]).

More recently, genomic data from ascarids and their hosts were also used to elucidate the coevolution between host and parasites [50]. Three ascarids from the giant panda, the red panda, and the lion were sequenced (Table 1) and used to construct a genome-wide phylogenetic tree. The topology of the tree for these ascarids based on the genome-wide data was not consistent with their host phylogeny, indicating that these parasites have not phylogenetically coevolved with their hosts [50]. However, analysis of the host–parasite protein–protein interactions (PPIs) reveals that seven of the PPIs, including the host neutrophil matrix metalloproteinase-8 (MMP8) and parasite lysosomal cysteine protease cathepsin Z (CTSZ), the host insulin-like growth factor-binding protein 7 (IGFBP7) and parasite prolyl 4-hydroxylase subunit beta (P4HB), and the host acute-phase protein (CRP) and parasite secreted member of the phospholipase A2 (PLA2G1B), had consistent phylogenetic topology [50]. These coevolutionary PPIs are involved in immune regulation and thus they may be relevant to the immune response during the antagonistic coevolution between ascarids and their hosts.

## 9. Future Perspectives

Over the last decade, the research community has established high-quality genomes, comprehensive transcriptomes, small RNA data, as well as other epigenomic datasets for *Ascaris*. Many related ascarids have also been sequenced and compared (Table 1). These genomic resources provide a starting point for future research in ascarids genomics and biology. I predict three major areas that will continue to grow and benefit from these and future genomic studies.

First, the sequencing of new species and continuing improvement of the genome assemblies [119] will be enabled by technologies, such as long reads (PacBio and Nanopore) and chromosome conformation capture (Hi-C), as well as genome annotation using long reads (ISO-seq), nascent transcription (PRO-seq), ribosome profiling, and epigenomics (ChIP-seq) data. The high-quality data will enable in-depth and chromosomal views of many biological processes, as seen in the improved *Ascaris* genomes and transcriptomes [34,35,36,37,38,39,46]. Research areas that have not been extensively studied in ascarids include alternative splicing [26], lncRNAs, epigenomes, regulation of tissue-specific transcripts, the repertoire and role of repeats, and more. New emerging technologies, such as single-cell genomics [120,121,122] and in situ sequencing for RNAs [123] and the genome [124], may also be used in the future to study the high-resolution, heterogeneity, and spatial organizations of DNA and RNA in the cells. Continuing the development of computational tools, genome browsers, and databases specifically designed for nematodes, such as WormBase ParaSite [125], will facilitate data mining and discovery for biologists working on these parasites.

Second, the resources will help researchers interested in various areas of nematode basic biology to carry out extensive genomic and molecular studies. For example, one can now characterize the nature and timing of the DNA breaks for *Ascaris* DNA elimination using these genomic resources by methods, such as END-seq [126]. The completed genome also enables the study of DNA replication timing, nascent transcriptional landscape, R-loops [127,128], and the 3-D genome organization that may be involved in the DNA break sites recognition and/or break generation as well as many other processes. In addition, histone modification profiles using ChIP-seq would enable the quantification of epigenetic changes throughout spermatogenesis, oogenesis, fertilization, zygote development, embryogenesis, and larvae development. Finally, comparative genomics using more completed chromosomal assemblies will delineate the evolution of nematode sex chromosomes [78] as well as the evolutionary developmental biology of embryogenesis and other developmental processes across nematode species.

Third, *Ascaris* and other ascarids are parasites that cause important zoonotic diseases. The genomic resources will enable future genome-scale studies on the epidemiology, population genomics, host–parasite interactions, metagenomics, and immune response of the diseases. High-quality genomes have now been used for extensive population genetics, linkage mapping, and genome-wide quantitative trait loci (QTLs) to examine disease transmissions, drug resistance, and mining for new therapeutic targets in parasitic worms (see recent reviews [129,130,131,132]). There is increasing evidence for drug resistance in parasitic nematodes, including ascarids [133,134]. *Ascaris* and other intestinal worm control programs are currently based on recurrent drug treatment [135,136,137] known to lead to drug resistance in nematodes, such as *Parascaris*. Thus, there is an urgency to identify and characterize new drug targets in parasitic nematodes. Future large-scale efforts using population and comparative genomics of major parasitic worms, such as the ones led by the International Helminth Genomes Consortium [47], are likely to identify new potential drug targets and screen compounds to combat parasitic worm infections.

## Figures and Tables

**Table 1:** Current genome assemblies for *Ascaris* and its relatives.

Features	*Ascaris suum*	*Ascaris lumbricoides*	*Parascaris univalens*	*Toxocara canis*	*Baylisascaris schroederi*	*Baylisascaris ailuri*	*Toxascaris leonina*
Major host	pig	human	horse	dog	giant panda	red panda	lion
Assembled bases (Mb)	279	296	253	317	282	267	285
N50 (kb)	12,191	4633	1826	375	889	51	36
Scaffold number	109 *	415	1274	22,857	2834	30,943	49,543
Largest scaffold (Mb)	23.1	13.2	5.6	1.9	5.8	0.5	0.4
Protein-coding genes	15,714	17,902	15,027	18,596	13,284	12,252	16,087
Accession number	JACCHR01	SMSY01	NJFU01	JPKZ01	NA	NA	NA
Major technologies used	PacBio, Hi-C	Illumina, PacBio	Illumina, PacBio, BioNano	Illumina	Illumina, PacBio	Illumina	Illumina
Reference	Wang et al. 2020 *Curr. Biol.* [39]	Easton et al. 2020 *eLife* [11]	Wang et al. 2017 *Genome Res.* [38]	Zhu et al. 2015 *Nat. Commun.* [49]	Hu et al. 2020 *Mol. Biol. Evol.* [50]		

* With 24 chromosomes, a mitochondrial genome, and 84 unplaced small contigs.

## Data Availability

Genomics and transcriptomes data for most ascarids are available in WormBase ParaSite (https://parasite.wormbase.org/species.html) (accessed on 27 March 2021) [125]. The NCBI accession numbers for the genome assemblies are available in Table 1 and Table S1. The *Ascaris* genome is also available in UCSC Genome Browser track data hubs [138] that can be access with this link: http://genome.ucsc.edu/s/jianbinwang/Ascaris_genome_browser_genes_review (accessed on 27 March 2021). The genome, transcripts and proteomes datasets for various ascarids are also available in https://dnaelimination.utk.edu/protocols-data/ (accessed on 27 March 2021).

## Supplementary Materials

The following are available online at https://www.mdpi.com/article/10.3390/genes12040493/s1, Table S1: History of *Ascaris* genome assemblies.
